# DeepVesselNet: Vessel Segmentation, Centerline Prediction, and Bifurcation Detection in 3-D Angiographic Volumes

**DOI:** 10.3389/fnins.2020.592352

**Published:** 2020-12-08

**Authors:** Giles Tetteh, Velizar Efremov, Nils D. Forkert, Matthias Schneider, Jan Kirschke, Bruno Weber, Claus Zimmer, Marie Piraud, Björn H. Menze

**Affiliations:** ^1^Department of Computer Science, TU München, München, Germany; ^2^Institute of Pharmacology and Toxicology, University of Zurich, Zurich, Switzerland; ^3^Department of Radiology, University of Calgary, Calgary, AB, Canada; ^4^Neuroradiology, Klinikum Rechts der Isar, TU München, München, Germany; ^5^Department for Quantitative Biomedicine, University of Zurich, Zurich, Switzerland

**Keywords:** vascular network, cross-hair filters, deepvesselnet, bifurcation, vessel segmentation, centerline, class balancing, vascular tree

## Abstract

We present DeepVesselNet, an architecture tailored to the challenges faced when extracting vessel trees and networks and corresponding features in 3-D angiographic volumes using deep learning. We discuss the problems of low execution speed and high memory requirements associated with full 3-D networks, high-class imbalance arising from the low percentage (<3%) of vessel voxels, and unavailability of accurately annotated 3-D training data—and offer solutions as the building blocks of DeepVesselNet. First, we formulate 2-D orthogonal cross-hair filters which make use of 3-D context information at a reduced computational burden. Second, we introduce a class balancing cross-entropy loss function with false-positive rate correction to handle the high-class imbalance and high false positive rate problems associated with existing loss functions. Finally, we generate a synthetic dataset using a computational angiogenesis model capable of simulating vascular tree growth under physiological constraints on local network structure and topology and use these data for transfer learning. We demonstrate the performance on a range of angiographic volumes at different spatial scales including clinical MRA data of the human brain, as well as CTA microscopy scans of the rat brain. Our results show that cross-hair filters achieve over 23% improvement in speed, lower memory footprint, lower network complexity which prevents overfitting and comparable accuracy that does not differ from full 3-D filters. Our class balancing metric is crucial for training the network, and transfer learning with synthetic data is an efficient, robust, and very generalizable approach leading to a network that excels in a variety of angiography segmentation tasks. We observe that sub-sampling and max pooling layers may lead to a drop in performance in tasks that involve voxel-sized structures. To this end, the DeepVesselNet architecture does not use any form of sub-sampling layer and works well for vessel segmentation, centerline prediction, and bifurcation detection. We make our synthetic training data publicly available, fostering future research, and serving as one of the first public datasets for brain vessel tree segmentation and analysis.

## 1. Introduction

Angiography offers insights into blood flow and conditions of the vascular tree. Three dimensional volumetric angiography information can be obtained using magnetic resonance (MRA), ultrasound, or x-ray based technologies like computed tomography (CT). A common first step in analyzing these data is vessel segmentation. Still, moving from raw angiography images to vessel segmentation alone might not provide enough information for clinical use, and other vessel features like centerline, diameter, or bifurcations of the vessels are also needed to accurately extract information about the vascular tree, for example, to characterize its structural properties or flow pattern. In this work, we present a deep learning approach, called DeepVesselNet, to perform vessel segmentation, centerline prediction, and bifurcation detection tasks. We make the code available (Tetteh, 2019a), and a ready-to-use implementation is available as companion material to our study “Machine learning analysis of whole mouse brain vasculature” (Todorov et al., 2020). DeepVesselNet deals with challenges that result from speed and memory requirements, unbalanced class labels, and the difficulty of obtaining well-annotated data for curvilinear volumetric structures by addressing the following three key limitations.

Processing 3-D medical volumes poses a memory consumption and speed challenge. Using 3-D convolutional neural networks (CNNs) leads to drastic increase in number of parameters to be optimized and computations to be executed when compared to 2-D CNNs. At the same time, applying a 2-D CNN in a slice-wise fashion discards valuable 3-D context information that is crucial for tracking curvilinear structures in 3-D. Inspired by the ideas of Rigamonti et al. (2013), Roth et al. (2014), and Liu et al. (2017) who proposed separable filters and used intersecting 2-D planes, we demonstrate the use of cross-hair filters from three intersecting 2-D filters, which helps to avoid the memory and speed problems of classical 3-D networks, while at the same time making use of 3-D information in volumetric data. Unlike the existing ideas where 2-D planes are extracted at a pre-processing stage and used as input channels (see discussion in section 2.1.2), our cross-hair filters are implemented on a layer level which help to retain the 3-D information throughout the network (see section 2.1).

The vessel, centerline and bifurcation prediction tasks is characterized by high class imbalances. Vessels account for <3% of the total voxels in a patient volume, centerlines represent a fraction of the segmented vessels, and visible bifurcations are in the hundreds at best—even when dealing with volumes with 10^6^ and more voxels. This bias toward the background class is a common problem in medical data (Grzymala-Busse et al., 2004; Christ et al., 2016; Haixiang et al., 2017). Unfortunately, current class balancing loss functions for training CNNs turn out to be numerically unstable in extreme cases as ours. We offer a solution to this “extreme class imbalance” problem by introducing a new loss function (see section 2.2) that we demonstrate to work well with our vascular features of interest.

Manually annotating vessels, centerlines, and bifurcations requires many hours of work and expertise. To this end, we demonstrate the successful use of simulation based frameworks (Szczerba and Székely, 2005; Schneider et al., 2012, 2014) that can be used for generating synthetic data with accurate labels (see section 2.3) for pre-training our networks, rendering the training of our supervised classification algorithm feasible. The transfer learning approach turns out to be a critical component for training CNNs in a wide range of angiography tasks and applications ranging from CT micrographs to TOF MRA. The synthesized and the clinical MRA datasets are made available publicly for future research and validation purposes. Further description and download link is provided in section 3.1.

### 1.1. Prior Work and Open Challenges

#### 1.1.1. Vessel Segmentation

Vessel enhancement and segmentation is a longstanding task in medical image analysis (see reviews by Kirbas and Quek, 2004; Lesage et al., 2009). The range of methods employed for vessel segmentation reflect the development of image processing during the past decades, including region growing techniques (Martínez-Pérez et al., 1999), active contours (Nain et al., 2004), statistical and shape models (Chung and Noble, 1999; Young et al., 2001; Liao et al., 2013; Moreno et al., 2013), particle filtering (Florin et al., 2006; Wörz et al., 2009; Dalca et al., 2011), and path tracing (Wang et al., 2013). All of these examples are interactive, starting from a set of seed labels as root and propagating toward the branches. Other approaches aim at an unsupervised enhancement of vascular structures: a popular multi-scale second order local structure of an image (Hessian) was examined by Frangi et al. (1998) with the purpose of developing a vessel enhancement filter. A measure of vessel-likeliness is then obtained as a function of all eigenvalues of the Hessian. A novel curvilinear structure detector, called Optimally Oriented Flux (OOF) was proposed by Law and Chung (2008) to find an optimal axis on which image gradients are projected to compute the image gradient flux. OOF has a lower computational load than the calculation of the Hessian matrix proposed in Frangi et al. (1998). A level-set segmentation approach with vesselness-dependent anisotropic energy weights is presented and evaluated in Forkert et al. (2013, 2011) for 3-D time-of-flight (TOF) MRA. Phellan and Forkert (2017) presented a comparative analysis of the accuracy gains in vessel segmentation generated by the use of nine vessel enhancement algorithms on time-of-flight MRA that included multi scale vesselness algorithms, diffusion-based filters, and filters that enhance tubular shapes and concluded that vessel enhancement algorithms do not always lead to more accurate segmentation results compared to segmenting non-enhanced images directly. An early machine learning approach for vessel segmentation was proposed by Schneider et al. (2015), combining joint 3-D vessel segmentation and centerline extraction using oblique Hough forest with steerable filters. In a similar fashion, Ciresan et al. (2012) used deep artificial neural network as a pixel classifier to automatically segment neuronal structures in stacks of electron microscopy images, a task somewhat similar to vessel segmentation. One example using deep learning architecture is the work of Phellan et al. (2017) who used a deep convolutional neural network to automatically segment the vessels of the brain in TOF MRA by extracting manually annotated bi-dimensional image patches in the axial, coronal, and sagittal directions as an input to the training process. Koziński et al. (2018) proposed a loss function that accommodates ground truth annotations of 2-D projections of the training volumes, for training deep neural networks in tasks where obtaining full 3-D annotations is a challenge.

Though deep learning has been applied in many medical imaging tasks, there are no dedicated architectures so far for vessel segmentation in 3-D volumetric datasets. Existing architectures might be sub-optimal and not work directly out of the box due to the unique nature of the vasculature as compared to other imaging tasks. There is therefore the need to explore other architectures and training strategies.

#### 1.1.2. Centerline Prediction

**Figure 1:**
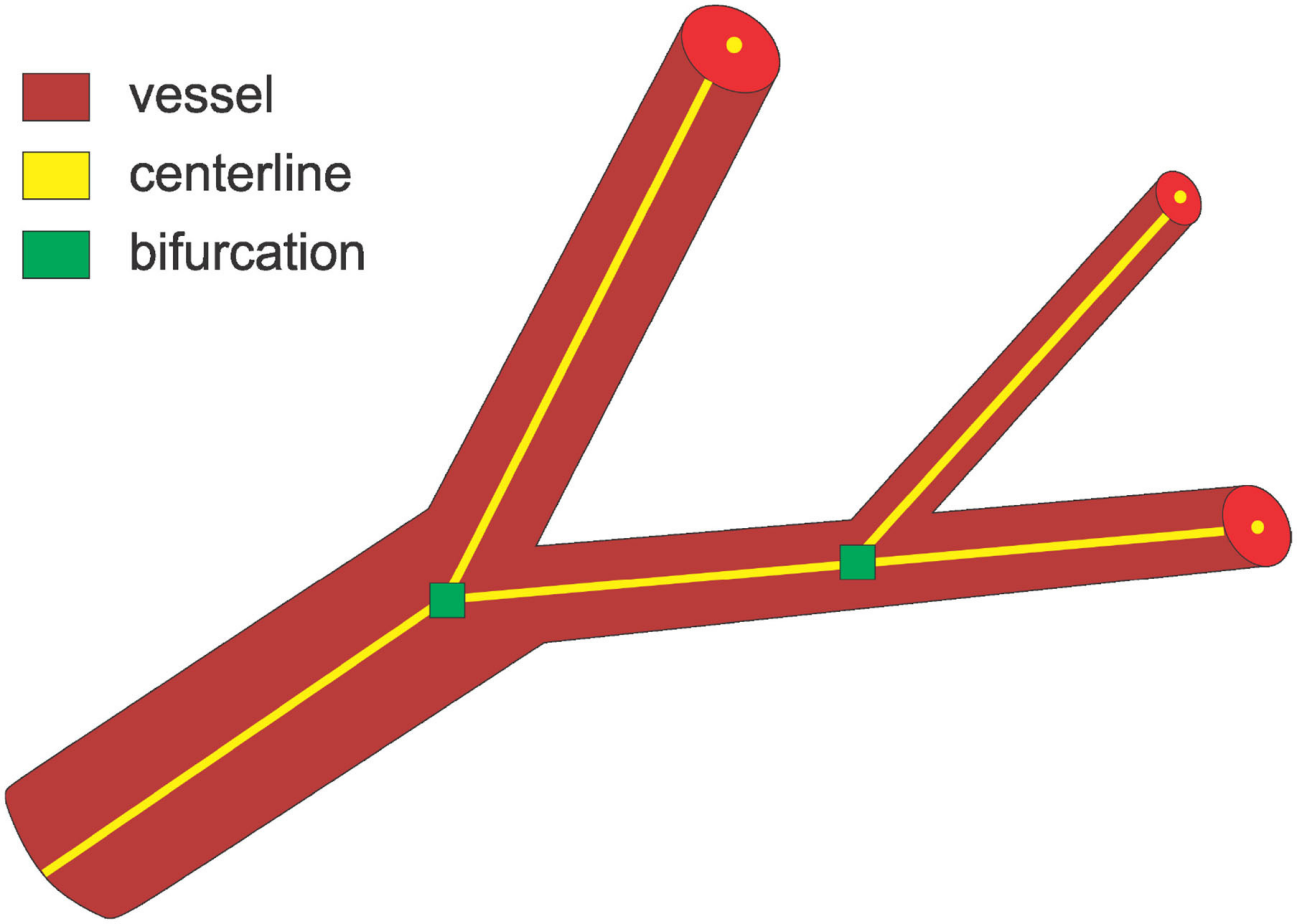
An overview of the three main tasks tackled in this paper. For bifurcations, we predict a neighborhood cube around the indicated point.

Identifying the center of a vessel is relevant for calculating the vessel diameter, but also for obtaining the “skeleton” of a vessel when extracting the vascular tree or network (see Figure 1). The vessels' skeleton and center can be found by post-processing a previously generated vessel segmentation. A method based on morphological operations is developed by Shagufta et al. (2014) which performs erosion using 2 × 2 neighborhoods of a pixel to determine if a pixel is a centerline candidate. Active contour models are applied in Maddah et al. (2003) as well as path planning and distance transforms for extracting centerline in vessels, and Chen and Cohen (2015) proposed a geodesic or minimal path technique. A morphology-guided level set model is used in Santamaría-Pang et al. (2007) to performed centerline extraction by learning the structural patterns of a tubular-like object, and estimating the centerline of a tubular object as the path with minimal cost with respect to outward flux in gray level images. Vesselness filters were adopted by Zheng et al. (2012) to predict the location of the centerline, while Macedo et al. (2010) used Hough transforms in handling a similar task. A Hough random forest with local image filters is designed in Schneider et al. (2015, 2012) to predict the centerline, and trained on centerline data previously extracted using one of the level set approaches.

The application of deep learning to the extraction of vessel centerline has not been explored. One reason may be the lack of annotated data necessary to train deep architectures that is hard to obtain especially in 3-D datasets.

#### 1.1.3. Bifurcation Detection

A vessel bifurcation refers to the point on a vessel centerline where the vessel splits into two vessels (see Figure 1). Bifurcations represent the nodes of the vascular tree or network and knowing their locations is important both for network extraction and for studying its properties (Rempfler et al., 2015). They represent structures that can easily be used as landmarks in image registration, but also indicate the locations of modified blood flow velocity and pressure within the network itself (Chaichana et al., 2017). Bifurcations are hard to detect in volumetric data as they are rare point-like features that vary in size and shape significantly. Similar to centerline extraction, the detection of bifurcations often happens by post-processing a previously generated vessel segmentation or by searching a previously extracted vessel graph. A two staged deep learning architecture is proposed in Zheng et al. (2015) for detecting carotid artery bifurcations as a specific landmark in volumetric CT data by first training a shallow network for predicting candidate regions followed by a sparse deep network for final prediction. A three stage algorithm for bifurcation detection is proposed in Chaichana et al. (2017) for digital eye fundus images, a 2-D task, and their approach included image enhancement, clustering, and searching the graph for bifurcations.

The direct predicting of the location of centerlines and bifurcations without a previous segmentation of vessels as an intermediate step is a task which has not been attempted yet. We foresee that having directly predicted centerlines and bifurcations together with those from postprocessing vessel segmentations will enhance the overall robustness and accuracy of the analysis of angiographic volumes.

## 2. Methodology

In the design of our DeepVesselNet architecture, we offer three methodological contributions: A. introducing fast cross-hair filters, B. dealing with extreme class balancing by relying on a loss function with stable weights, and C. generating synthetic 3D vessel structures for training DeepVesselNet and other standard segmentation architectures.

### 2.1. Cross-Hair Filters Formulation

**Figure 2:**
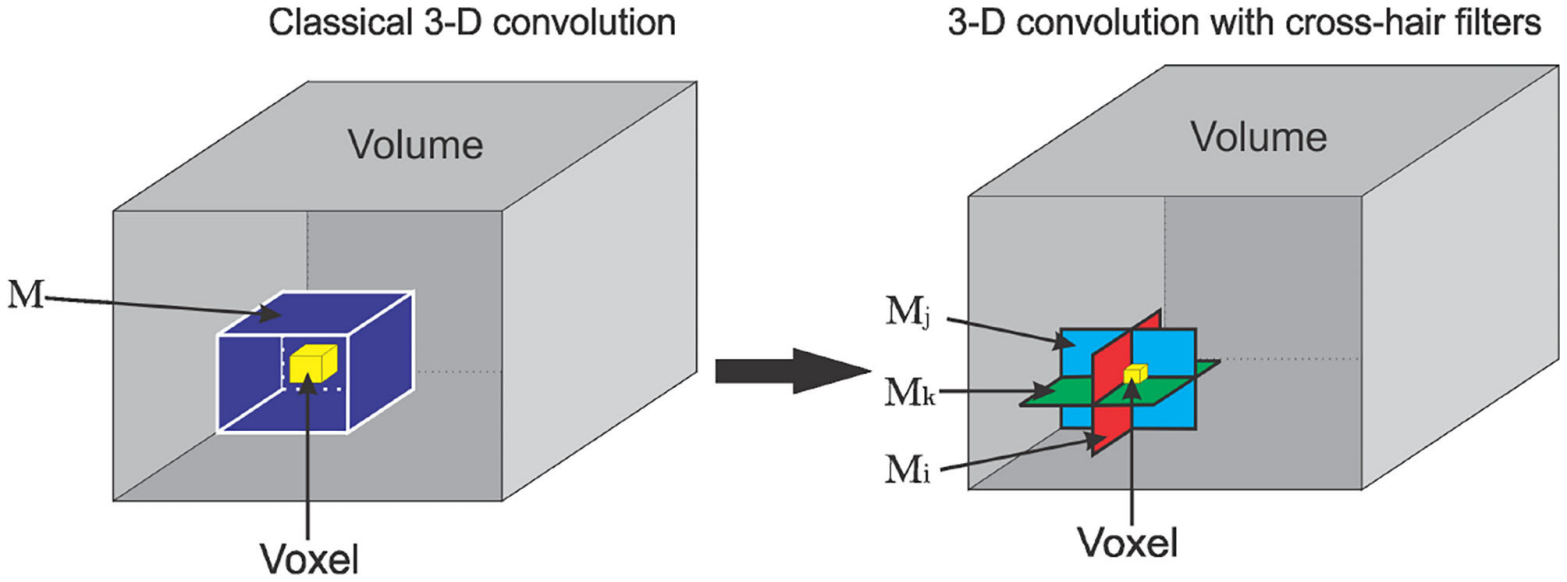
Graphical representation of cross-hair filters for 3-D convolutional operation. **(Left)** A classical 3-D convolution with filter *M*. **(Right)** Cross-hair 3-D convolutional with 2-D filter stack *M*_*i*_, *M*_*j*_, *M*_*k*_.

In this section, we introduce the 3-D convolutional operator, which utilizes cross-hair filters to improve speed and memory usage while maintaining accuracy. For a graphical representation of classical 3-D convolutional operator and the proposed cross-hair filters is see Figure 2. Let *I* be a 3-D volume, M a 3-D convolutional kernel of shape (*k*_*x*_, *k*_*y*_, *k*_*z*_), and ∗ be a convolutional operator. We define ∗ as:

(1) $$\begin{array}{l} I*M=A=\left\{a_{ijk}\right\};\;a_{ijk}=\overset{k_{x}}{\underset{r=1}{\sum }}\overset{k_{y}}{\underset{s=1}{\sum }}\overset{k_{z}}{\underset{t=1}{\sum }}I_{\left(R,S,T\right)}M_{\left(r,s,t\right)}; \end{array}$$

(2) $$\begin{array}{l} R=i+r-\left(1+\left[\frac{k_{x}}{2}\right]\right), \end{array}$$

(3) $$\begin{array}{l} S=j+s-\left(1+\left[\frac{k_{y}}{2}\right]\right),\\ T=k+t-\left(1+\left[\frac{k_{z}}{2}\right]\right), \end{array}$$

where {*a*_*ijk*_} is a position element of matrix *A*, *I*_(*R,S,T*)_ is the intensity value of image *I* at voxel position (*R, S, T*), *M*_(*r,s,t*)_ is the value of kernel *M* at position (*r, s, t*), and [*c*] is the greatest integer less or equal to *c*.

From Equation (1), we see that a classical 3-D convolution involves *k*_*x*_*k*_*y*_*k*_*z*_ multiplications and *k*_*x*_*k*_*y*_*k*_*z*_−1 additions for each voxel of the resulting image. For a 3 × 3 × 3 kernel, we have 27 multiplications and 26 additions per voxel. Changing the kernel size to 5 × 5 × 5 increases the complexity to 125 multiplications and 124 additions per voxel. This then scales up with the dimension of the input image. For example, a volume of size 128 × 128 × 128 and a 5 × 5 × 5 kernel results in about 262 × 10^6^ multiplications and 260 × 10^6^ additions. To handle this increased computational complexity, we approximate the standard 3-D convolution operation by

(4) $$\begin{array}{l} a_{ijk}=\overset{k_{y}}{\underset{s=1}{\sum }}\overset{k_{z}}{\underset{t=1}{\sum }}I_{\left(i,S,T\right)}M_{\left(s,t\right)}^{i}+\overset{k_{x}}{\underset{r=1}{\sum }}\overset{k_{z}}{\underset{t=1}{\sum }}I_{\left(R,j,T\right)}M_{\left(r,t\right)}^{j}\\ \;+\overset{k_{x}}{\underset{r=1}{\sum }}\overset{k_{y}}{\underset{s=1}{\sum }}I_{\left(R,S,k\right)}M_{\left(r,s\right)}^{k}, \end{array}$$

where *M*^*i*^, *M*^*j*^, *M*^*k*^ are 2-D convolutional (cross-hair) kernels used as an approximation to the 3-D kernel *M* in (1) along the *i*th, *j*th, and *k*th axes, respectively. *R, S*, and *T* are as defined in (1). Using cross-hair filters results in (*k*_*y*_*k*_*z*_ + *k*_*x*_*k*_*z*_ + *k*_*x*_*k*_*y*_) multiplications and (*k*_*y*_*k*_*z*_ + *k*_*x*_*k*_*z*_ + *k*_*x*_*k*_*y*_ − 1) additions. If we let *k*_*m*1_, *k*_*m*2_, *k*_*m*3_ be the sizes of the kernel *M* such that *k*_*m*1_ ≥ *k*_*m*2_ ≥ *k*_*m*3_, we can show that

(5) $$\begin{array}{l} k_{y}k_{z}+k_{x}k_{z}+k_{x}k_{y}\leq 3\left(k_{m1}k_{m2}\right)\leq k_{x}k_{y}k_{z}, \end{array}$$

where strict inequality holds for all *k*_*m*3_ > 3. Equation (5) shows a better scaling in speed and also in memory since the filters sizes in (1) and (4) are affected by the same inequality. With the approximation in (4), and using the same example as above (volume of size 128 × 128 × 128 and a 5 × 5 × 5 kernel), we now need <158 × 10^6^ multiplications and 156 × 10^6^ additions to compute the convolution leading to a reduction in computation by more than 100 × 10^6^ multiplications and additions when compared to a classical 3-D convolution. Increasing the volume or kernel size, further increases the gap between the computational complexity of (1) and (4). Moreover, we will see later from our experiments that (4) still retains essential 3-D context information needed for the classification task.

#### 2.1.1. Efficient Implementation

**Figure 3:**
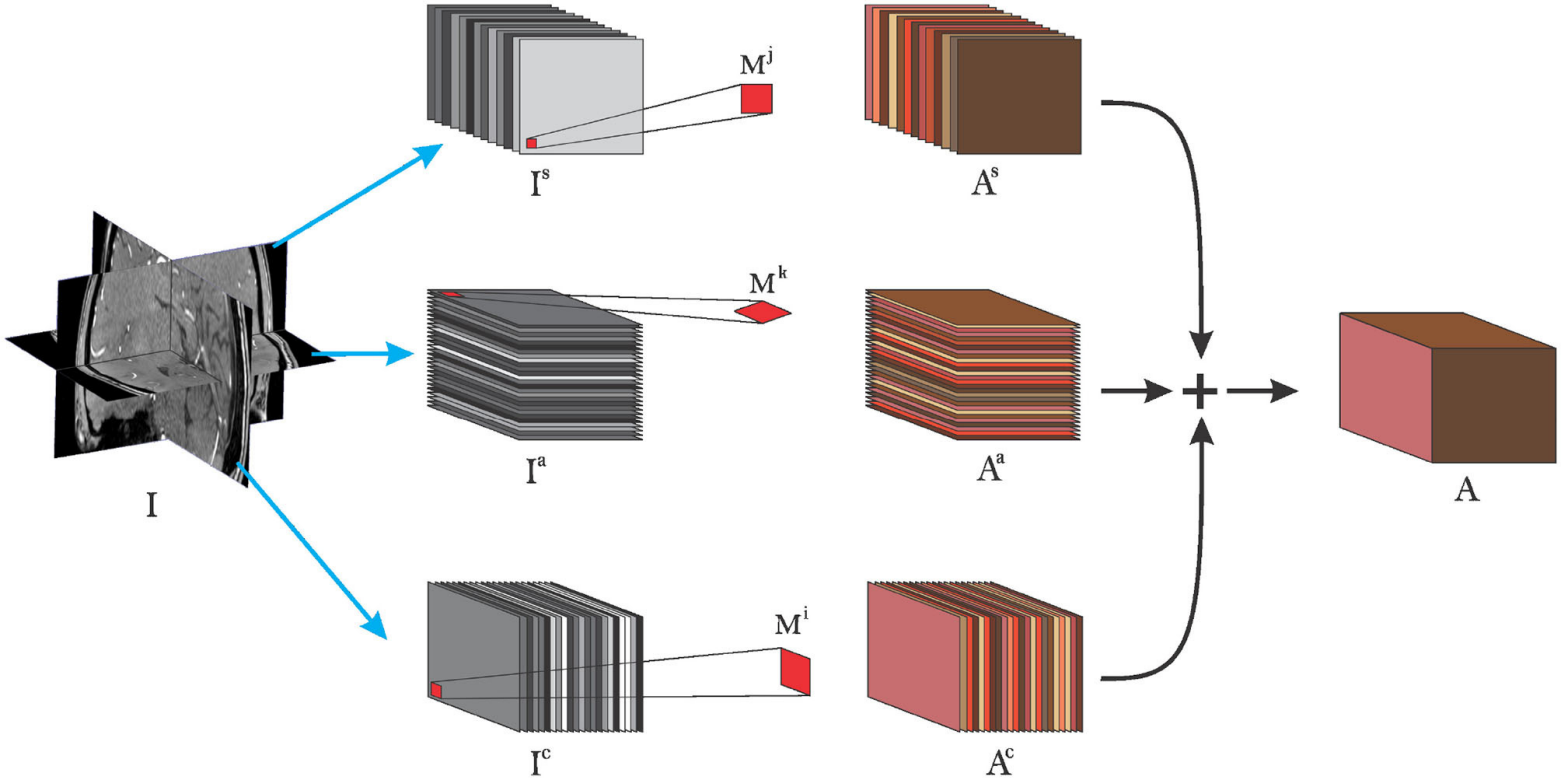
Pictorial view of efficient implementation of cross-hair filters. Grayscaled stacks refer to input to the layer, red shaped squares refer to 2-D kernels used for each plane. Brown colored slices refer to extracted features after convolution operations and + symbol refers to matrix addition.

In Equation (4), we presented our 2-D crosshair filters. However, applying (4) independently for each voxel leads to a redundant use of memory. More precisely, voxels close to each other share some neighborhood information and making multiple copies of it is not memory efficient. To this end we present an efficient implementation below (depicted in Figure 3). Consider *I* as defined in Equation (1) and let us extract the sagital, coronal, and axial planes as *I*^*s*^, *I*^*c*^, and *I*^*a*^, respectively. By application of Equations (1) and (4), we have a final implementation as follows:

(6) $$\begin{array}{l} I◇M=A=\beta_{c}A^{c}+\beta_{s}A^{s}+\beta_{a}A^{a},\\ \;A^{c}=I^{c}**M^{i},\;A^{s}\;=\;I^{s}**M^{j},\;A^{a}\;=\;I^{a}**M^{k}, \end{array}$$

where ∗∗ refers to a 2-D convolution along the first and second axes of the left matrix over all slices in the third axis. β_*c*_, β_*s*_, and β_*a*_ are weights to control the input of the planes toward the final sum, for example, in the case of different spatial resolutions of the planes (we use β_*c*_ = β_*s*_ = β_*a*_ = 1 in our experiments) and ◇ refers to our crosshair filter operation. This implementation is efficient in the sense that it makes use of one volume at a time instead of copies of the volume in memory where voxels share the same neighborhood. In other words, we still have only one volume in memory but rather rotate the kernels to match the slices in the different orientations. This lowers the memory requirements during training and inference, allowing to train on more data with little memory.

#### 2.1.2. 2.5-D Networks vs. 3-D Networks With Cross-Hair Filters

Its important to discuss the difference between existing 2.5-D networks and our proposed cross-hair filters. Given a 3-D task (e.g., vessel segmentation in 3-D volume), a 2.5-D based network handles the task by considering one 2-D slice at a time. More precisely, the network takes a 2-D slice (sometimes with few neighboring slices) as input and classifies all pixels in this slice. This is repeated for each slice in the volume and the final results from the slices are fused again to form the 3-D result. On the architecture level, 2.5-D networks are 2-D networks with a preprocessing method for extracting 2-D slices and a postprocessing method for fusing 2-D results into a 3-D volume. We note that the predictions of 2.5-D networks are solely based on 2-D context information. Examples of 2.5-D networks are the implementation of UNet in Christ et al. (2016) used for liver and lesion segmentation tasks in CT volumetric dataset, and the network architecture of Sekuboyina et al. (2017) for annotation of lumbar vertebrae. Extensions of this approach may include a pre-processing stage where several 2-D planes are extracted and used as input channels to the 2-D network (Roth et al., 2014; Liu et al., 2017).

On the other hand, 3-D networks based on our proposed cross-hair filters take the whole 3-D volume as input, and at each layer in the network we apply the convolutional operator discussed in section 2.1. Therefore, our filters make use of 3-D context information at each convolutional layer and do not require specific preprocessing or post processing. Our proposed method differs from classical 3-D networks in the sense that it uses less parameters and memory since it does not use full 3-D convolutions. However, it is worth noting that our filters scale exactly the same as 2.5-D (i.e., in only two directions) with respect to changes in filter and volume sizes. More precisely, given a square or cubic filter of size *k*, we have *k*^2^ parameters in a 2.5-D network and 3*k*^2^ in our cross-hair filter based network. Increasing the filter size by a factor of *r* will scale up as *k* + *r* quadratically in both situations [i.e., (*k* + *r*)^2^ for 2.5-D and 3(*k* + *r*)^2^ in cross-hair filter case] as compared to full 3-D networks where the parameter size scales as a cube of *k* + *r*.

In summary, unlike the existing 2.5-D ideas where 2-D planes are extracted at a pre-processing stage and used as input channels to a 2-D network architecture, our cross-hair filters are implemented on a layer level which help retain the 3-D information throughout the network making it a preferred option when detecting curvilinear objects in 3-D.

### 2.2. Extreme Class Balancing With Stable Weights

We now discuss the problem of “extreme” class imbalance and introduce a new cost function that is capable of dealing with this problem. Often in medical image analysis, the object of interest (e.g., vessel, tumor etc.) accounts for a minority of the total voxels of the image. The objects of interest in the datasets used in this work account for <2.5% of the voxels (the different datasets are described in section 3.1). A standard cross entropy loss function is given by

(7) $$\begin{array}{l} L\left(\text{W}\right)=-\frac{1}{N}\overset{N}{\underset{j=1}{\sum }}y_{j}\log P\left(y_{j}=1|X;\text{W}\right)+\left(1-y_{j}\right)\\ \;\log\left[1-P\left(y_{j}=1|X;\text{W}\right)\right], \end{array}$$

$$\begin{array}{l} L\left(\text{W}\right)=-\frac{1}{N}(\underset{j\in Y_{+}}{\sum }\log P\left(y_{j}=1|X;\text{W}\right)\\ \;+\underset{j\in Y_{-}}{\sum }\log P\left(y_{j}=0|X;\text{W}\right)), \end{array}$$

where *N* is the total number of examples, *P* is the probability of obtaining the ground truth label given the data *X* and network weights **W**, *y*_*j*_ is the label for the *j*th example, *X* is the feature set, *W* is the set of parameters of the network, *Y*_+_ is the set of positive labels, and *Y*_−_ is the set of negative (background) labels. Using this cost function with extreme class imbalance between *Y*_−_ and *Y*_+_ could cause the training process to be biased toward detecting background voxels at the expense of the object of interest. This normally results in predictions with high precision against low recall. To remedy this problem, Hwang and Liu (2015) proposed a biased sampling loss function for training multiscale convolutional neural networks for a contour detection task. This loss function introduced additional trade-off parameters and it samples twice more edge patches than non-edge ones for positive cost-sensitive finetuning, and vice versa, for negative cost-sensitive finetuning. Based on this, Xie and Tu (2015) proposed a class-balancing cross entropy loss function of the form

(8) $$\begin{array}{l} L\left(\text{W}\right)=-\beta \underset{j\in Y_{+}}{\sum }\log P\left(y_{j}=1|X;\text{W}\right)\\ \;-\left(1-\beta \right)\underset{j\in Y_{-}}{\sum }\log P\left(y_{j}=0|X;\text{W}\right), \end{array}$$

where **W** denotes the standard set of parameters of the network, which are trained with backpropagation and β and 1 − β are the class weighting multipliers, which are calculated as $\beta =\frac{\left|Y_{-}\right|}{\left|Y\right|}$, $1-\beta =\frac{\left|Y_{+}\right|}{\left|Y\right|}$. *P*(.) is the probability from the final layer of the network, and *Y*_+_ and *Y*_−_ are the set of positive and negative class labels, respectively.

#### 2.2.1. Challenges From Numerical Instability and High False Positive Rate

The idea of giving more weight to the cost associated with the class with the lowest count from Equation (8), has been used in other recent works (Christ et al., 2016; Maninis et al., 2016; Nogues et al., 2016; Roth et al., 2016). However, our experiments (in section 3.4) show that the above loss function raises two main challenges.

First, there is the problem of numerical instability. The gradient computation is numerically unstable for very big training sets due to the high values taken by the loss. More precisely, there is a factor of $\frac{1}{N}$, that scales the final sum to the mean cost in the standard cross-entropy loss function in Equation (7). This factor ensures that the gradients are stable irrespective of the size of the training data *N*. However, in Equation (8), the weights β and 1 − β do not scale the cost to the mean value. For high number of data points |*Y*| (which is usually the case of voxel-wise tasks), the sums explode leading to numerical instability. For example, given a perfectly balanced data, we have β = 1 − β = 0.5, irrespective of the number of data points |*Y*|. Thus, increasing the size of the dataset (batch size) has no effect on the weights (β) but increases the number of elements in the summation, causing the computations to be unstable.

Second, there are challenges from high false positive rate. A high rate of false positives leading to high recall values is observed during training and at test time. This is caused by the fact that in most cases the object of interest accounts for <5% of the total voxels (about 2.5% in our case). Therefore, we have a situation where 1 − β < 0.05, which implies that wrongly predicting 95 background voxels as foreground is less penalized in the loss than predicting 5 foreground voxels as background. This leads to high false positive rate and, hence, high recall values.

#### 2.2.2. A New “Extreme” Class Balancing Function

To address the challenges discussed above, we introduce different weighting ratios and an additional factor to take care of the high false positive rate; and define:

(9) $$\begin{array}{l} L\left(\text{W}\right)=L_{1}\left(\text{W}\right)+L_{2}\left(\text{W}\right)\\ L_{1}\left(\text{W}\right)=-\frac{1}{\left|Y_{+}\right|}\underset{j\in Y_{+}}{\sum }\log P\left(y_{j}=1|X;\text{W}\right)\\ \;-\frac{1}{\left|Y_{-}\right|}\underset{j\in Y_{-}}{\sum }\log P\left(y_{j}=0|X;\text{W}\right)\\ L_{2}\left(\text{W}\right)=-\frac{\gamma_{1}}{\left|Y_{+}\right|}\underset{j\in Y_{f+}}{\sum }\log P\left(y_{j}=0|X;\text{W}\right)\\ \;-\frac{\gamma_{2}}{\left|Y_{-}\right|}\underset{j\in Y_{f-}}{\sum }\log P\left(y_{j}=1|X;\text{W}\right)\\ \gamma_{1}=0.5+\frac{1}{\left|Y_{f+}\right|}\underset{j\in Y_{f+}}{\sum }|P\left(y_{j}=0|X;\text{W}\right)-0.5|\\ \gamma_{2}=0.5+\frac{1}{\left|Y_{f-}\right|}\underset{j\in Y_{f-}}{\sum }|P\left(y_{j}=1|X;\text{W}\right)-0.5| \end{array}$$

where *Y*_*f*+_ and *Y*_*f*−_ are the set of false positive and false negative predictions respectively and |.| is the cardinality operator which measures the number of elements in the set. $L_{1}$ is a more numerically stable version of Equation (8) since it computes the voxel-wise, cost which scales well with the size of the dataset or batch. But the ratio of β to 1 − β is maintained as desired. $L_{2}$ [false prediction (FP) Rate Correction] is introduced to penalize the network for false predictions. However, we do not want to give false positive (*Y*_*f*+_) and false negatives (*Y*_*f*−_) the same weight as total predictions (*Y*_+_,*Y*_−_), since we will end up with a loss function without any class balancing because the weights will offset each other. Therefore, we introduce γ_1_ and γ_2_, which depend on the mean absolute distance of the wrong predicted probabilities from 0.5 (the value can be changed to suit the task). This allows us to penalize false predictions, which are very far from the central point (0.5). The false predictions (*Y*_*f*+_, *Y*_*f*−_) are obtained through a 0.5 probability threshold. Experimental results from application of FP rate correction can be found in section 3.3.2.

### 2.3. Synthetic Data for Transfer Learning

**Figure 4:**
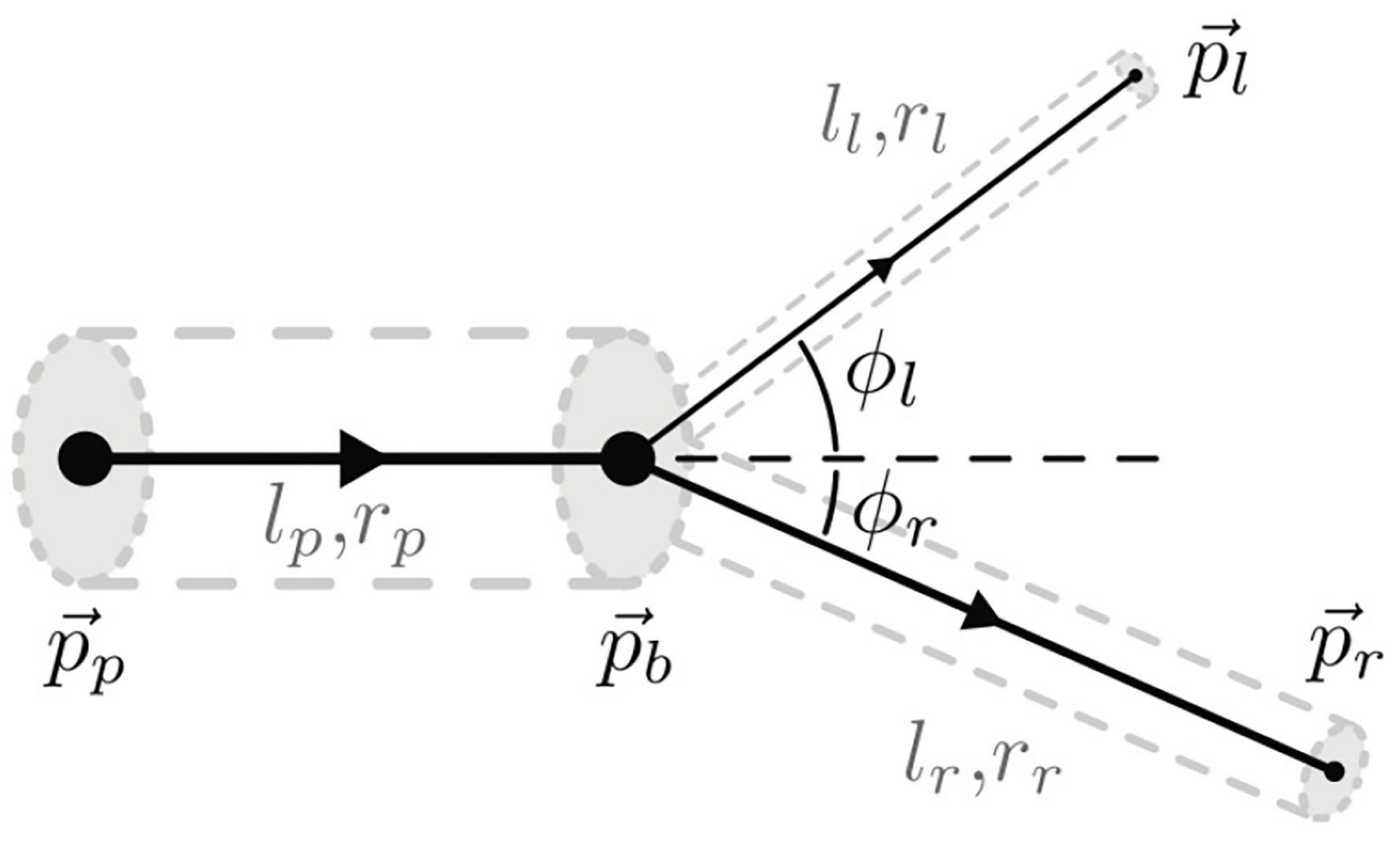
A representation of the constrained bifurcation configuration, as presented in Schneider et al. (2012), where *l*_*p*_, *l*_*r*_, and *l*_*l*_ are the length of the parent, right daughter, and left daughter segments, respectively. *p*_*r*_ and *p*_*l*_ are the right and left daughter nodes, respectively.

To generate synthetic data, we follow the method of Schneider et al. (2012) which implements a simulator of a vascular tree that follows a generative process inspired by the biology of angiogenesis. This approach, described in Schneider et al. (2012), has initially been developed to complement missing elements of a vascular tree, a common problem in μCT imaging of the vascular bed (Schneider et al., 2014). We now use this generator to simulate physiologically plausible vascular trees that we can use in training our CNN algorithms. The simulator considers the mutual interplay of arterial oxygen (*O*_2_) supply and vascular endothelial growth factor (VEGF) secreted by ischemic cells to achieve physiologically plausible results. Each vessel segment is modeled as a rigid cylindrical tube with radius *r* and length *l*. It is represented by a single directed edge connecting two nodes. Semantically, this gives rise to four different types of nodes, namely root, leaf, bifurcation, and inter nodes. Each node is uniquely identified by the 3-D coordinate $\vec{P}={\left(x,y,z\right)}^{T}$. Combining this with connectivity information, fully captures the geometry of the approximated vasculature. The tree generation model and the bifurcation configuration is shown in Figure 4. The radius of parent bifurcation branch *r*_*p*_, and the radius of left (*r*_*l*_) and right (*r*_*r*_) daughter branches are related by a bifurcation law (also known as Murray's law) given by $r_{p}^{\gamma }=r_{l}^{\gamma }+r_{r}^{\gamma }$, where γ is the bifurcation exponent. Our simulator enforces the Murray's law during the tree generation process. Further constraints, $\cos\left(\phi_{l}\right)=\frac{r_{p}^{4}+r_{l}^{4}-r_{r}^{4}}{2r_{p}^{2}r_{l}^{2}}$ and $\cos\left(\phi_{r}\right)=\frac{r_{p}^{4}+r_{r}^{4}-r_{l}^{4}}{2r_{p}^{2}r_{r}^{2}}$ are placed on the bifurcation angles of the left (ϕ_*l*_) and right (ϕ_*r*_) vessel extension elements respectively. This corresponds corresponds to the optimal position of the branching point ${\vec{P}}_{b}$ with respect to a minimum volume principle, another constraint enforced in the simulator from Schneider et al. (2012, 2014).

#### 2.3.1. Properties of the Simulated Data

The output of the generation process is a tree with information on the 3-D position $\vec{P}$ of the nodes, their type (root, bifircation, inter, leaf), and connectivity information, which includes the edge *E*_*ij*_ between two nodes *N*_*i*_ and *N*_*j*_, and its radius *R*_*ij*_. We reconstruct a 3-D volumetric data from this abstracted network description by modeling each vessel segment as a cylinder in 3-D space. We simulate different background and foreground intensity patterns with different signal-to-noise ratios. Detailed description of generated data is given in section 3.1.

## 3. Experiments, Results, and Discussion

### 3.1. Datasets

In this work, we use three different datasets to train and test the networks. In all three data sets, the test cases are kept apart from the training data and are used only for testing purposes. The datasets can be downloaded for public research from the paper's GitHub page (Tetteh, 2019a).

#### 3.1.1. Synthetic Dataset

**Figure 5:**
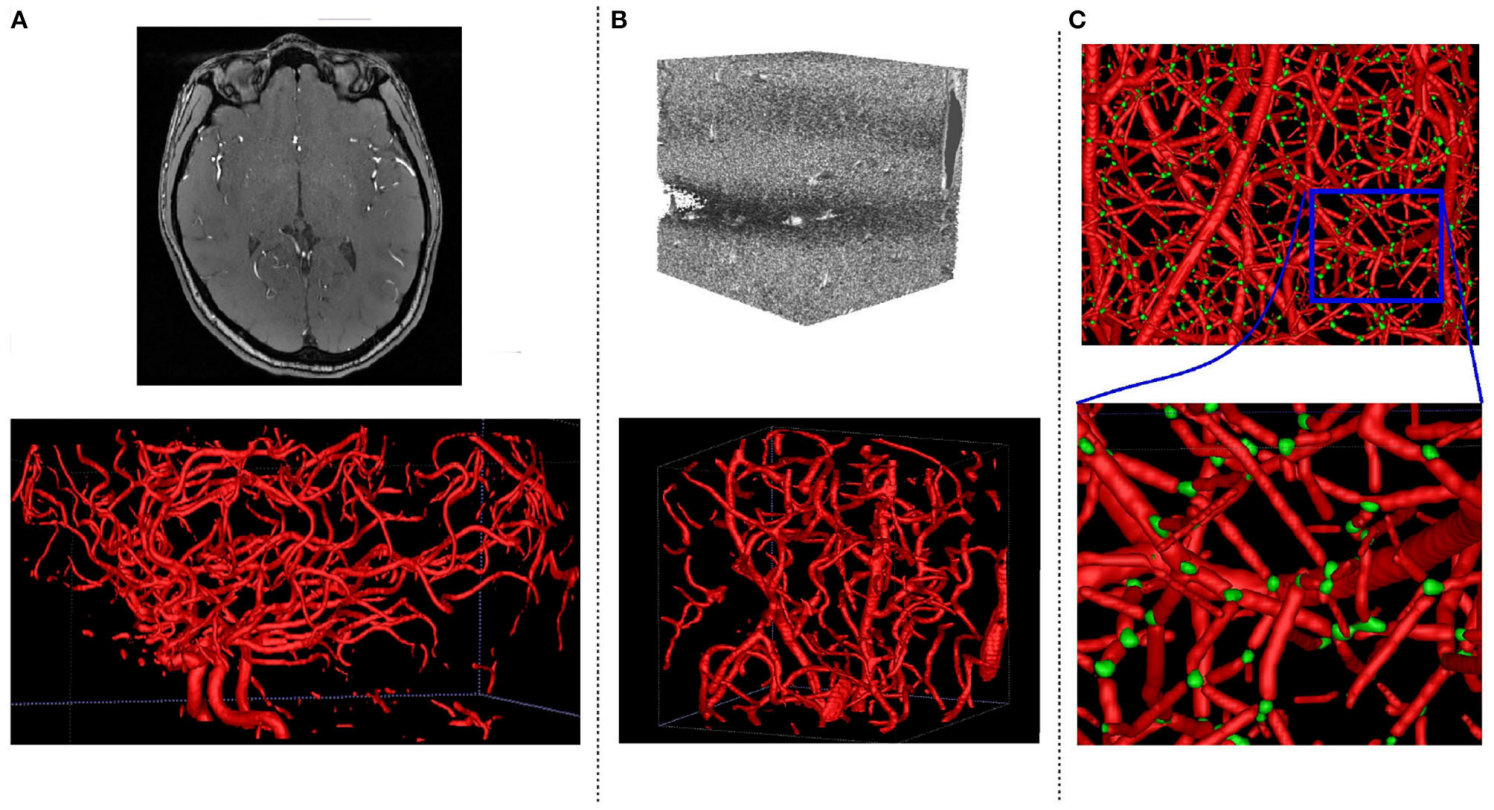
Sample of datasets used in our experiments with the corresponding ground truth segmentations. **(A)** Clinical MRA, **(B)** μCTA, **(C)** Synthetic.

Training convolutional networks from scratch typically requires significant amounts of training data. However, assembling a properly labeled dataset of 3-D curvilinear structures, such as vessels and vessel features, takes a lot of human effort and time, which turns out to be the bottleneck for most medical applications. To overcome this problem, we generate synthetic data based on the method proposed in Schneider et al. (2012, 2014). A brief description of this process has already been presented in section 2.3. In the arterial tree generation experiment, the parameters in Table 1 of Schneider et al. (2012) are used. We use the default (underlined) values for all model parameters. We initialize the processes with different random seeds and scale the resulting vessel sizes in voxels to match the sizes of vessels in clinical datasets. Vessel intensities are randomly chosen in the interval [128, 255] and non-vessel intensities are chosen from the interval [0 − 100]. Gaussian noise is then applied to the generated volume randomly changing the mean (i.e., in the range [−5, 5]) and the standard deviation (i.e., in the range [−15, 30]) for each volume. We generate 136 volumes of size 325 × 304 × 600 with corresponding labels for vessel segmentation, centerlines, and bifurcation detection. Vessel, centerline and bifurcation labels occupy 2.1, 0.2, and 0.05% of total intensities, respectively, further highlighting the problem of class imbalance. Twenty volumes out of the 136 is used as a test set and the remaining volumes are used for pre-training our networks in the various tasks at hand. An example of the synthetic dataset can be found in Figure 5C. The synthetic dataset including both training and test volumes with ground truth labels for vessel, centerlines, and bifurcation are available at Tetteh (2019b) for download and public use.

#### 3.1.2. Clinical Magnetic Resonance Angiograph (MRA) Dataset

To finetune and test our network architectures on real data, we obtain 40 volumes of clinical TOF MRA of the human brain, 20 of which are fully annotated, and the remaining 20 partially annotated using the method proposed by Forkert et al. (2013). Each volume has a size of 580 × 640 × 136 and spacial resolution of 0.3125 × 0.3125 × 0.6*mm* on the coronal, sagittal, and axial axes, respectively. We select 15 out of the 20 fully annotated volumes for testing and use the remaining five as a validation set. We also correct the 20 partially annotated volumes by manually verifying some of the background and foreground voxels. This leads to three labels, which are true foreground (verified foreground), true background (verified background), and the third class, which represent the remaining voxels not verified. In our later experiments, we use the true foreground and background labels to finetune our networks. This approach helps in avoiding any uncertainty with respect to using the partially annotated data for finetuning of the network. Image intensity ranges were scaled with a quadratic function to enhance bright structures and normalized to a standard range after clipping high intensities. A sample volume of the TOF MRA dataset can be found in Figure 5A.

#### 3.1.3. Micro Computed Tomogaphy Angiography (μCTA)

A 3-D volume of size 2, 048 × 2, 048 × 2, 740 and spacial resolution 0.7 × 0.7 × 0.7*mm* is obtained from synchrotron radiation X-ray tomographic microscopy of a rat brain. From this large volume, we extract a dataset of 20 non-overlaping volumes of size 256 × 256 × 256, which were segmented using the method proposed by Schneider et al. (2015), and use them in our later experiments to finetune the networks. To create a test set, we manually annotate 52 slices in 4 other volumes different from the 20 volumes above (208 slices in total). As with the clinical MRA data, image intensity ranges for the μCTA were also scaled with a quadratic function to enhance bright structures and normalized to a standard range after clipping high intensities. Detailed description of the μCTA data can be found in Reichold et al. (2009), and a sample volume is presented in Figure 5B.

### 3.2. Network Architecture and Implementations

In this study we focus on the use of artificial neural networks for the tasks of vessel segmentation, centerline prediction, and bifurcation detection. Different variants of state-of-the-art Fully Convolutional Neural Networks have been presented for medical image segmentation (Christ et al., 2016; Maninis et al., 2016; Milletari et al., 2016; Nogues et al., 2016; Roth et al., 2016; Sekuboyina et al., 2017; Tetteh et al., 2017). Most of these architectures were based on the popular idea of convolutional-deconvolutional network which applies down-sampling at the earlier layers of the network and then reconstruct the volume at the later layers through up-sampling. This may be a bad choice given that the vascular tree tasks, especially centerline prediction and bifurcation detection, require fine details at the voxel level which can easily be lost through down-sampling. We therefore use a fully convolutional network (FCN) without down-sampling and up-sampling layers as a preferred architecture to test the performance of DeepVesselNet discussed in sections 2.1, 2.2, and 2.3. Nonetheless we also implement DeepVesselNet with popular convolutional-deconvolutional architectures to systematically study the effect of cross-hair kernel, as well as training behavior and generalization. Python implementation of our cross-hair filters and all other codes used in our experiments is available on GitHub (Tetteh, 2019a) for public use.

#### 3.2.1. DeepVesselNet-FCN

**Figure 6:**
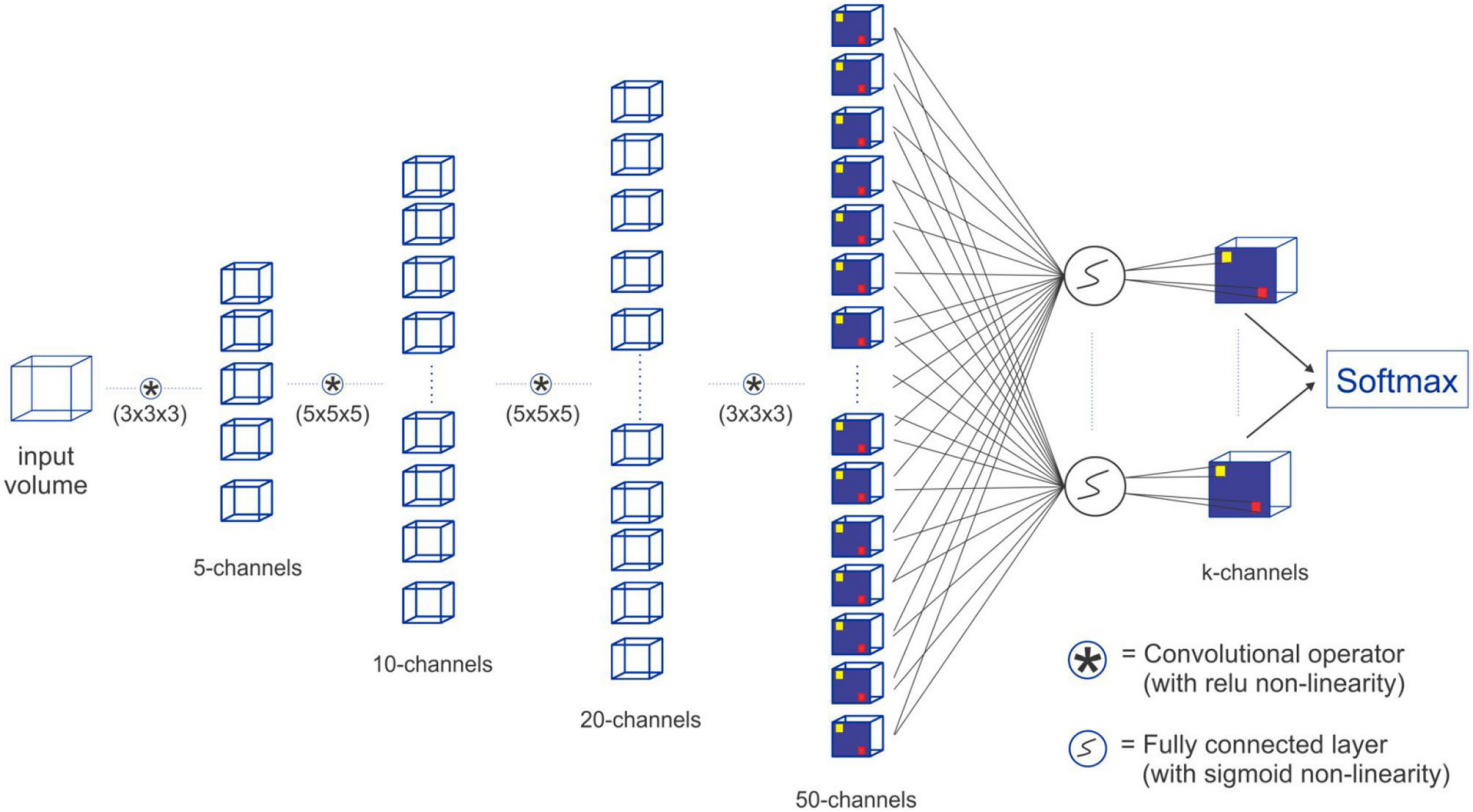
Our proposed DeepVesselNet-FCN architecture implementation with crosshair filters.

We construct a Fully Convolutional Network FCN with four convolutional layers and a sigmoid classification layer. In this implementation, we do not use down-sampling and up-sampling layers and we carry out the convolutions in a way that the output image is of the same size as the input image by zero-padding. The removal of the down-sampling and up-sampling layers is motivated by the fact that the tasks (vessel segmentation, centerline prediction, and bifurcation detection) involve fine detailed voxel sized objects and down-sampling has an effect of averaging over voxels which causes these fine details to be lost. The alternative max-intensity pooling can easily change the voxel position of the maximum intensity later in the up-sampling stage of the network. With DeepVesselNet-FCN implementation, we have a very simple 5-layer fully-convolutional network, which takes a volume of arbitrary size and outputs a segmentation map of the same size. For the network structure and a description of the parameters (see Figure 6).

#### 3.2.2. DeepVesselNet-VNet and DeepVesselNet-Unet

To analyse the properties of our proposed cross-hair filters, we implement two alternative convolutional-deconvolutional architectures—VNet (Milletari et al., 2016) and 3D UNet (Çiçek et al., 2016)—and replace all 3-D convolutions with our proposed cross-hair filters discussed in section 2.1 to obtain DeepVesselNet-VNet and DeepVesselNet-UNet, respectively. By comparing the parameter size and execution time of DeepVesselNet-VNet and DeepVesselNet-UNet to the original VNet and 3D UNet implementations, we can evaluate the improvement in memory usage as well as the gain in speed that cross-hair filters offer. We also use these implementations to test whether gain in speed and memory consumption have a significant effect on prediction accuracy. Finally, DeepVesselNet-VNet and DeepVessel-UNet architectures include sub-sampling (down-sampling and up-sampling) layers. By comparing these two architecture with DeepVesselNet-FCN we can evaluate the relevance of sub-sampling when handling segmentation of fine structures like vessels and their centerlines and bifurcations.

#### 3.2.3. Network Configuration, Initialization, and Training

We use the above described architecture to implement three binary networks for vessel segmentation, centerline prediction, and bifurcation detection. Network parameters are randomly initialized, according to the method proposed in Bengio and Glorot (2010), by sampling from a uniform distribution in the interval $\left(-\frac{1}{\sqrt{k_{x}k_{y}k_{z}}},\frac{1}{\sqrt{k_{x}k_{y}k_{z}}}\right)$ where (*k*_*x*_ × *k*_*y*_ × *k*_*z*_) is the size of the given kernel in a particular layer. For each volume in our training set, we extract non-overlapping boxes of size (64 × 64 × 64) covering the whole volume and then feed them through the network for the finetuning of parameters. The box extraction is only done at training time to enable fast training and efficient use of computation memory, this is however not needed after our convolutional kernels are trained since full volumes can be used at test time. We train the network using a stochastic gradient descent optimizer without regularization. During pre-training, we use a learning rate of 0.01 and decay of 0.99, which is applied after every 200 iterations for all network architectures. For finetuning, we use a learning rate of 0.001 and a decay of 0.99 applied after every 200 iterations. We implement our algorithm using the THEANO (Theano Development Team, 2016) Python framework and train on a machine with 64GB of RAM and Nvidia TITAN X 12GB GPU.

### 3.3. Evaluating the DeepVesselNet Components

Prior to evaluating the performance of DeepVesselNet, we conducted a series of experiments to test the components of DeepVesselNet which includes fast cross-hair filters, the FP rate correction, and pre-training on synthetic data. Results of this ablation analysis are offered here.

#### 3.3.1. Fast Cross-Hair Filters

To investigate the usefulness of cross-hair filters in DeepVesselNet, we experiment with full 3-D versions of DeepVesselNet and evaluate the effect on performance based on three main criteria—memory footprint based on number of parameters, computational speed based on execution time, and prediction accuracy based on Dice score. Table 1 shows the number of parameters in the various architectures and the execution times in the three datasets. Comparing DeepVesselNet-VNet and DeepVesselNet-UNet with their 3-D versions (VNet and UNet), we find more than 27% (16.56 vs. 22.89 m and 4.45 vs. 7.41 m, respectively) reduction in memory footprint. Also, the execution time in Table 1 shows that cross-hair filters improve the computational speed of DeepVesselNet-VNet and DeepVesselNet-UNet by more than 23% over VNet and UNet respectively in both synthetic and clinical MRA datasets. DeepVesselNet-FCN uses very low (only 0.05 m) number of parameters as compared to the other architectures due to the absence of sub-sampling layers. Scores in Table 1 are obtained using kernels of size 3 × 3 × 3 and 5 × 5 × 5, and the benefits of using sparse cross-hair filter will be even more profound with larger kernel sizes and volume sizes. Evaluation of cross-hair filters in terms of prediction accuracy is discussed in section 3.4.

**Table 1:** Number of convolutional parameters in the networks used in our experiments.

**Architecture**	**Params**	**Ex. time**	**Ex. time**	**Ex. time**
	**(millions)**	**Synthetic (s)**	**TOF MRA (s)**	**μCTA (s)**
DeepVesselNet-FCN	0.05	13	13	4
DeepVesselNet-VNet	16.56	17	20	7
DeepVesselNet-UNet	4.45	13	14	4
VNet	22.89	23	26	11
UNet	7.41	17	19	6

*For the purpose of comparison, the number of parameters stated here refers to only the convolutional layers in the various architectures. Ex. Time refers to the average time in seconds required to process one volume in the sythetic and MRA TOF datasets*.

#### 3.3.2. Extreme Class Balancing ($L_{1}+L_{2}$)

**Figure 7:**
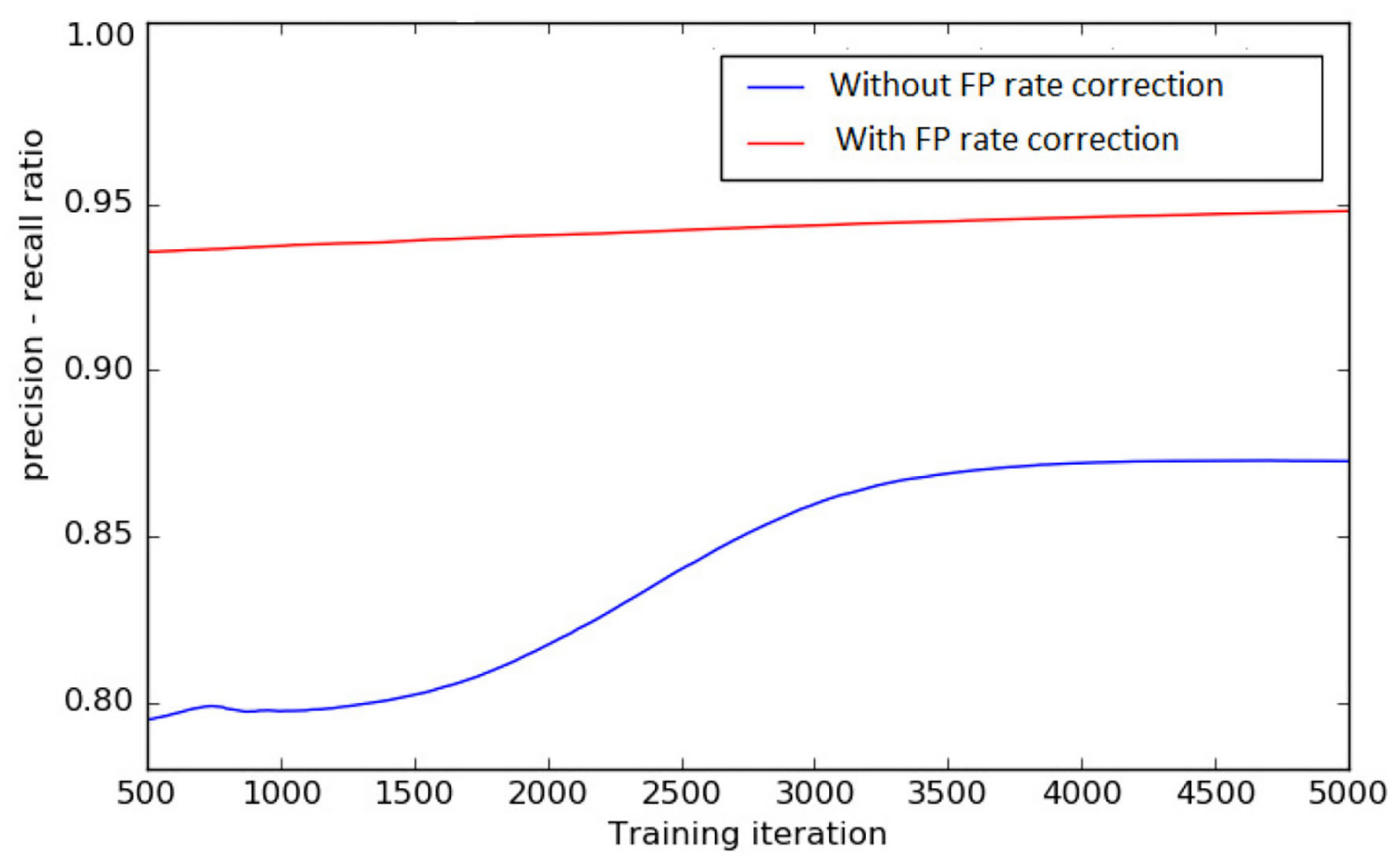
Precision—recall ratio during training, with FP rate correction and without FP rate correction in the loss function, on four selected clinical MRA volumes. A balanced precision-recall ratio (i.e., close to 1) implies that we obtain the FP rate correction we propose in the work and the training process is not bias toward the background or the foreground.

To test the effect of FP rate correction loss function discussed in section 2.2, we train the DeepVesselNet-FCN architecture on a sub-sample of four clinical MRA volumes from scratch, with and without FP rate correction described in Equation (9). We train for 5,000 iterations and record the ratio of precision to recall every 5 iterations using a threshold of 0.5 on the probability maps. A plot of the precision-recall ratio during training without FP rate correction ($L_{1}$ Only) and with FP rate correction ($L_{1}+L_{2}$) is presented in Figure 7. The results of this experiments suggest that training with both factors in the loss function, as proposed in section 2.2, keeps a better balance between precision and recall (i.e., a ratio closer to 1.0) than without the second factor. A balanced precision-recall ratio implies that the training process is not bias toward the background or the foreground. This helps prevent over-segmentation, which normally occurs as a result of the introduction of the class balancing.

#### 3.3.3. Transfer Learning From Synthetic Data

We assess the usefulness of transfer learning with synthetic data by comparing the training convergence speed, and various other scores that we obtain when we pretrain DeepVesselNet-FCN on synthetic data and finetune on the clinical MRA dataset, compared to training DeepVesselNet-FCN from scratch on the clinical MRA. For this experiment, we only consider the vessel segmentation task, as no annotated clinical data is available for centerline and bifurcation tasks. Results of this experiment are reported in Table 2. We achieve a Dice score of 86.39% for training from scratch without pre-training on synthetic data and 86.68% when pretraining on synthetic data. This shows that training from scratch or pre-training on synthetic data does not make much difference regarding the accuracy of the results. However, training from scratch requires about 600 iterations more than pre-training on synthetic data for the network to converge (i.e., 50% more longer).

**Table 2:** Results from pretraining DeepVesselNet-FCN on synthetic data and finetuning with the training set from the clinical MRA vs. training DeepVesselNet-FCN from scratch on clinical MRA.

**Method**	**Precision**	**Recall**	**Dice**	**Iterations**
With pretraining	86.44	86.93	86.68	1200
Without pretraining	85.87	86.92	86.39	1800

*Iterations refers to training iterations required for the network to converge. Although the result in Dice score are not very different, it is clear that the pre-training on synthetic data leads to an earlier convergence of the network*.

### 3.4. Evaluating DeepVesselNet Performance

In this subsection, we retain the best training strategy from the described experiments in section 3.3 and assess the performance of our proposed network architecture with other available methods mainly on the vessel segmentation task. As a further validation of our methodology we handle centerline prediction and bifurcation detection using the proposed architectures. Given a good vessel segmentation, centerline prediction, and bifurcation detection tasks is classically handled by applying vessel skeletonization as a post processing step and a search of the resulting graph. Our aim in applying our architecture to handle these tasks is not to show superiority over the existing vessel skeletonization methods but it is to serve as a further verification of the effects of our described methodology and to offer a complementary way of obtaining centerlines and bifurcations, for example, to increase the robustness of the processing pipeline when fusing results of complementary approaches. The details of these experiments, results and discussion are given below.

#### 3.4.1. Vessel Segmentation

**Figure 8:**
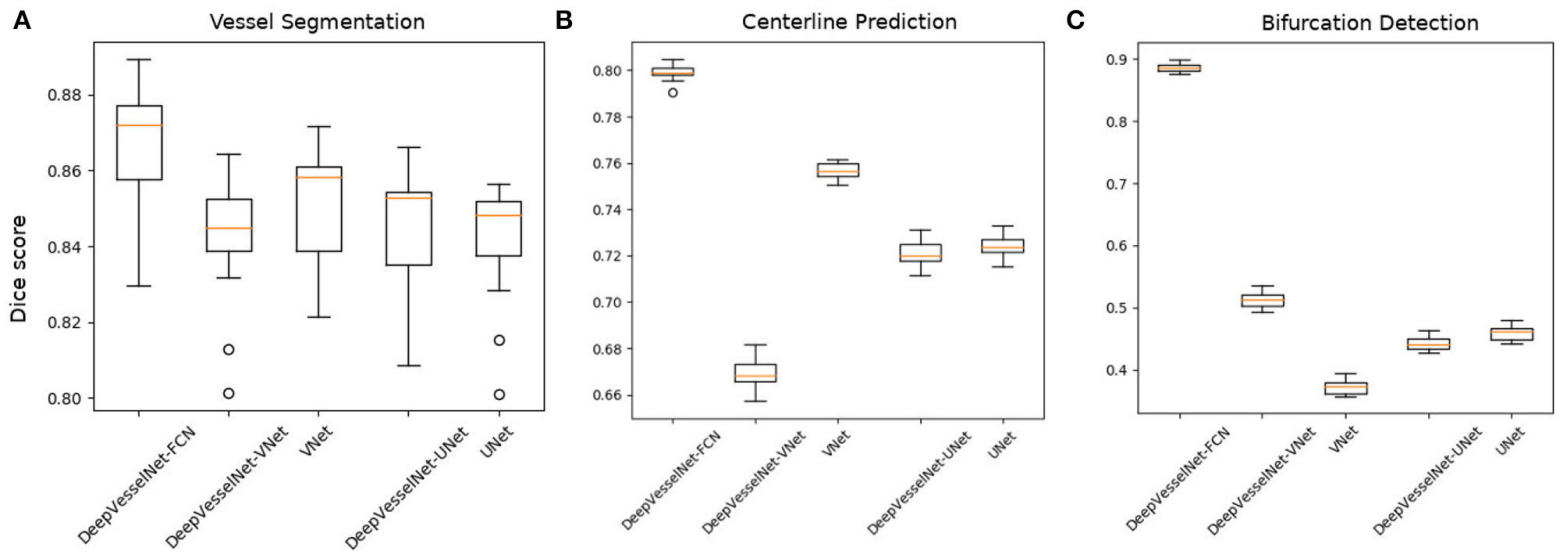
Box plots of Dice scores from vessel segmentation **(A)**, centerline prediction **(B)**, and bifurcation detection **(C)** tasks over our test set in the clinical MRA dataset across the deep learning architectures. It is evident that DeepVesselNet-VNet and DeepVesselNet-UNet obtain comparable results as VNet and UNet, respectively. However, DeepVesselNet-FCN achieves a significantly higher results in all three tasks as confirmed by a *p* < 0.001.

We pretrain DeepVesselNet-(FCN, VNet, UNet) architectures on synthetic volumes for vessel segmentation and evaluate its performance on TOF MRA volumes through a transfer learning approach. We later finetune the networks with additional clinical MRA data, repeating the evaluation. Table 3 reports results of these tests, together with performances of competing methods. We obtain a Dice score of 81.48% for DeepVesselNet-FCN, 81.32% for DeepVessel-UNet, and 80.10% for DeepVesselNet-VNet on TOF MRA test dataset with the transfer learning step, and 86.68% (DeepVesselNet-FCN), 84.36% (DeepVesselNet-UNet) as well as 84.25% (DeepVesselNet-VNet) after finetuning. This results (also box plots in Figure 8) suggest that, with a Cox-Wilcoxon significance test *p* < 0.001, DeepVesselNet-FCN which does not use sub-sampling outperforms the versions of networks that use sub-sampling layers (VNet and UNet). Table 3 also reports results from the methods of Schneider et al. (2015) and Forkert et al. (2013) all of which are outperformed by DeepVesselNet-FCN in terms of Dice score. Comparing DeepVesselNet-VNet and VNet (84.25 vs. 84.97% with a *p*-value of 0.04) as well as DeepVesselNet-Unet and UNet (84.36 vs. 84.68 with a *p*-value of 0.07) on the MRA data, we find an advantage of up to 1% for the latter in terms of Dice scores. However, DeepVesselNet-VNet and DeepVesselNet-Unet have the advantage of being memory and computationally efficient as seen in Table 1. These results show that cross-hair filters can be used in DeepVesselNet at a little to no cost in terms of vessel segmentation accuracy.

**Table 3:** Results for vessel segmentation.

**Dataset**	**Method**	**Prec**.	**Rec**.	**Dice**
Synthetic	DeepVesselNet-FCN	**99.84**	**99.87**	**99.86**
	DeepVesselNet-VNet	99.54	99.59	99.56
	DeepVesselNet-UNet	99.48	99.42	99.45
	VNet	99.48	99.50	99.49
	UNet	99.57	99.52	99.55
	Schneider et al.	99.47	99.56	99.52
TOF MRA	DeepVesselNet-FCN (finetuned)	**86.44**	**86.93**	**86.68**
	DeepVesselNet-FCN (pretrained)	82.76	80.25	81.48
	DeepVesselNet-VNet (finetuned)	85.00	83.51	84.25
	DeepVesselNet-VNet (pretrained)	83.32	77.12	80.10
	DeepVesselNet-UNet (finetuned)	83.56	85.18	84.36
	DeepVesselNet-UNet (pretrained)	83.48	79.27	81.32
	VNet (finetuned)	84.34	85.62	84.97
	VNet (pretrained)	82.41	75.82	78.98
	UNet (finetuned)	84.02	85.35	84.68
	UNet (pretrained)	83.16	80.23	81.67
	Schneider et al.	84.81	82.15	83.46
	Forkert et al.	84.99	73.00	78.57
μCTA	DeepVesselNet-FCN	**96.72**	95.82	**96.27**
	DeepVesselNet-VNet	95.83	**96.18**	96.01
	DeepVesselNet-UNet	95.85	96.06	95.95
	VNet	95.25	95.84	95.55
	UNet	95.27	95.71	95.49
	Schneider et al.	95.15	91.51	93.30

*TOF MRA are evaluated within the brain region only*.

*Pretrained results refers to the scores we obtained on the test set after pretraining, and finetuned results are scores after finetuning with annotated data available for TOF-MRA*.

*Best performing method in each metric are show in bold*.

#### 3.4.2. Centerline Prediction

**Figure 9:**
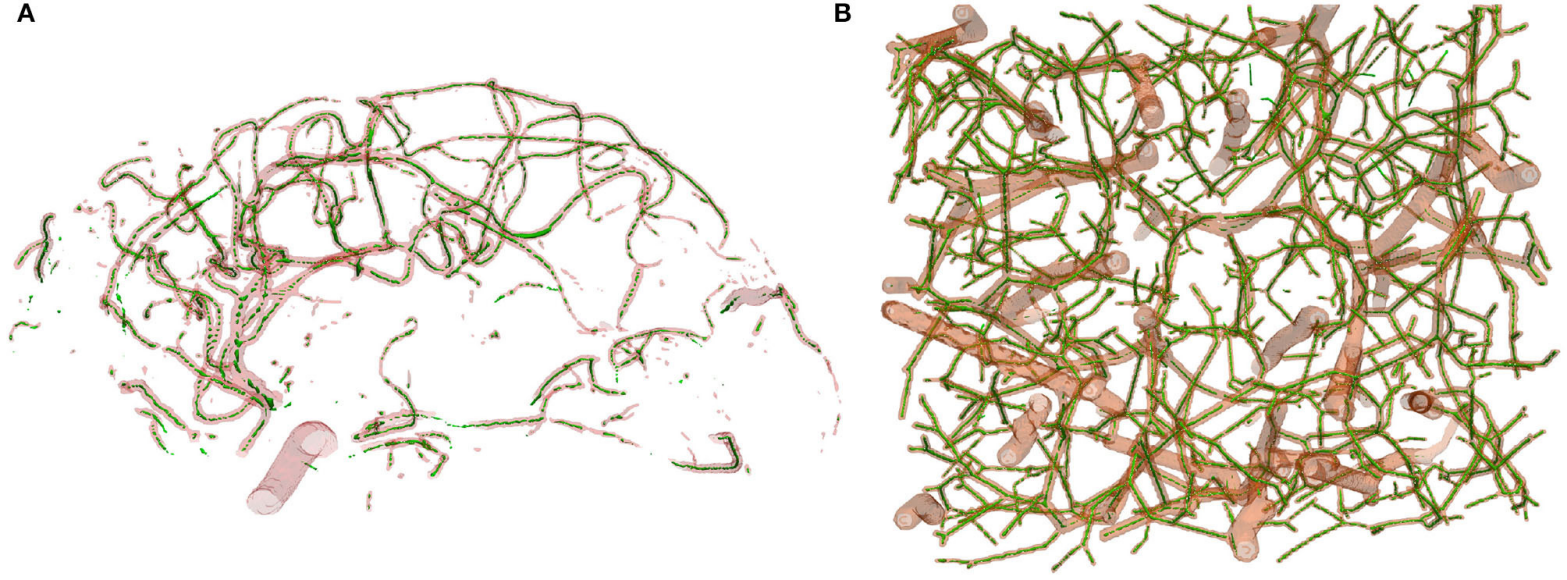
Centerline prediction on MRA TOF **(A)** and Synthetic **(B)** test datasets using DeepVesselNet-FCN (centerline in green). There are more detections in smaller vessels than in larger vessels which can be explained by the network seeing more smaller vessels than bigger vessels during training. Again, centerline detection in MRA TOF covers all the vessels with missing points and can be improved by finetuning on annotated MRA data or by a post-processing strategy to fill in the missing points.

For centerline prediction, we train DeepVesselNet on the synthetic dataset, test it on synthetic dataset and present visualizations on synthetic and clinical MRA datasets (see Figure 9). The networks use the probabilistic segmentation masks from the vessel segmentation step as an input. Qualitative results are presented in Figure 9 together with quantitative scores in Table 4. DeepVesselNet-VNet performs slightly worse than VNet in terms of the Dice score (66.96 vs. 74.82% with a *p*-value of 0.0001). Similar trend can be seen when we compare Dice scores of DeepVesselNet-UNet and UNet (72.10 vs. 72.41% with a *p*-value of 0.0001). We obtain a Dice score of 79.92% for DeepVesselNet-FCN, which outperforms UNet and VNet and their corresponding DeepVesselNet variants with a significance test *p* < 0.0001. Here we note that the morphological operations based method of Schneider et al., which represents a state of the art method for centerline prediction, is able to obtain a higher recall than DeepVesselNet-FCN method (86.03 vs. 82.35%). This means that it detects more of the centerline points than DeepVesselNet-FCN. However, it suffers from lower precision (48.07 vs. 77.63%) due to higher false positive rate which causes the overall performance to fall (61.68 vs. 79.92% Dice score) as compared to DeepVesselNet-FCN. From the box plots in Figure 8 it is very evident DeepVesselNet-FCN significantly outperforms all other architectures suggesting that the performance of the other architectures suffers from the use of sub-sampling layers.

**Table 4:** Results for centerline prediction tasks.

**Method**	**Prec**.	**Rec**.	**Dice**
DeepVesselNet-FCN	**77.63**	82.35	**79.92**
DeepVesselNet-VNet	65.15	68.87	66.96
DeepVesselNet-UNet	71.28	72.95	72.10
VNet	76.41	73.30	74.82
UNet	71.25	73.61	72.41
Schneider et al.	48.07	**86.03**	61.68

*Results suggest that architectures with sub-sampling layers suffer fall in performance due to loss of fine details which is crucial in centerline prediction*.

*Best performing methods in each category in bold*.

#### 3.4.3. Bifurcation Detection

**Figure 10:**
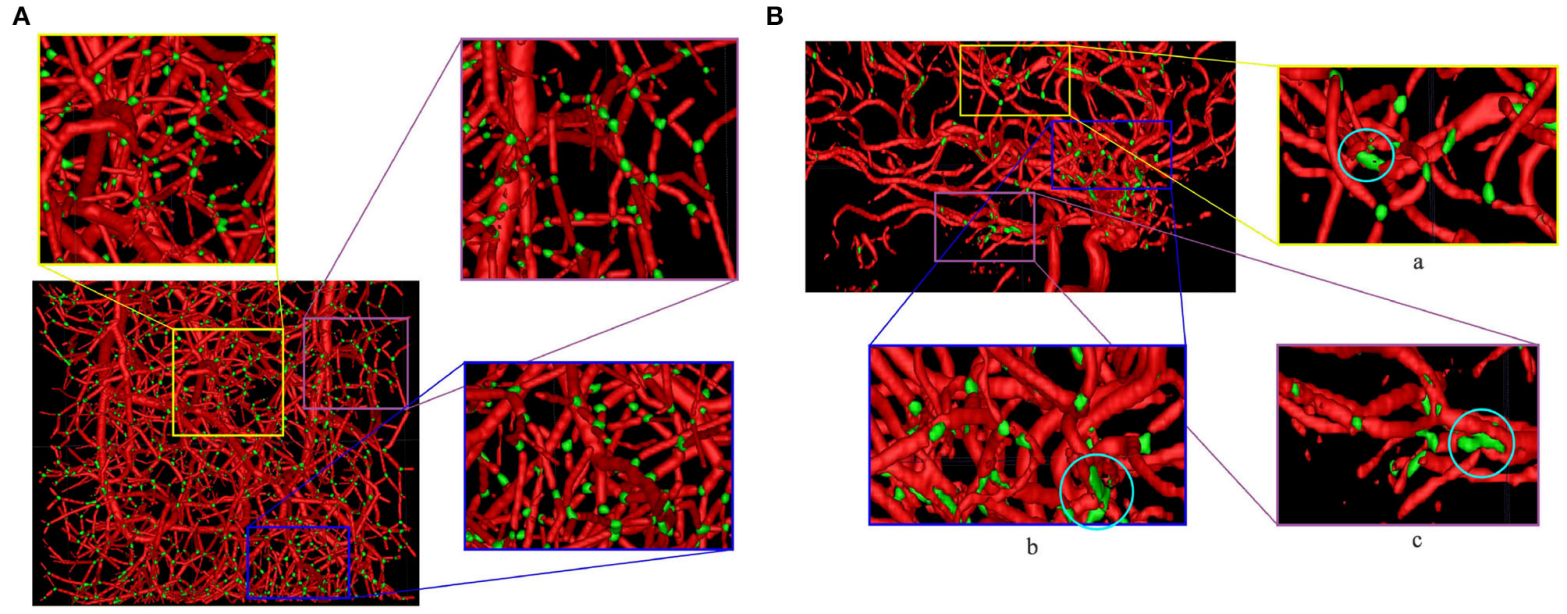
Bifurcation detection on synthetic **(A)** and MRA TOF **(B)** test datasets using DeepVesselNet-FCN (bifurcations in green). Similar to centerline prediction, bifurcation detections in smaller vessels are better than in bigger vessels which might be due to the network seeing more examples in smaller vessels than in bigger vessels during training. At regions where a lot of vessels intersect, the network predicts it as a big bifurcation, this can be seen in the circled regions in zoomed images (a–c).

For a quantitative evaluation of DeepVesselNet in bifurcation detection, we use synthetically generated data, and adopt a two-input-channels strategy. We use the vessel segmentations as one input channel and the centerline predictions as a second input channel relying on the same training and test splits as in the previous experiments. In our predictions we aim at localizing a cubic region of size (5 × 5 × 5) around the bifurcation points, which are contained within the vessel segmentation. We evaluate the results based on a hit-or-miss criterion: a bifurcation point in the ground truth is counted as hit if a region of a cube of size (5 × 5 × 5) centered on this point overlaps with the prediction, and counted as a miss otherwise; a hit is considered as true positive (TP) and a miss is considered as false negative (FN); a positive label in the prediction is counted as false positive (FP) if a cube of size (5 × 5 × 5) centered on this point contains no bifurcation point in the ground truth. Qualitative results on synthetic and clinical MRA TOF are shown in Figure 10, respectively. Results for Schneider et al. are obtained by first extracting the vessel tree and searching the graph for nodes. Then all nodes with two or more splits are treated as bifurcations—this being one of the standard methods for bifurcation extraction. In Figure 8, we present the box plots of Dice score distributions obtained by the different architectures over our test set. Results from Table 5 and Figure 8 show that DeepVesselNet-FCN performs better than the other architectures in 5 out of 6 metrics. In our experiments, it became evident that VNet tends to over-fit, possibly due to its high number of parameters. This may explain why results for VNet are worse than all other methods, also suggesting that in cases where little training data is available, the DeepVesselNet-FCN architecture may be the preferable due to low number of parameters and the absence of sub-sampling layers.

**Table 5:** Results from bifurcation detection experiments.

**Method**	**Prec**.	**Rec**.	**Det. %**	**Mean err**	**Err std**
DeepVesselNet-FCN	**78.80**	**92.97**	**86.87**	0.2090	**0.6671**
DeepVesselNet-VNet	46.80	56.70	84.21	1.6533	0.9645
DeepVesselNet-UNet	29.47	88.41	85.89	0.6227	0.9380
VNet	25.50	68.71	70.29	1.2434	1.3857
UNet	32.57	77.81	71.78	1.2966	1.4000
Schneider et al.	77.18	85.08	84.30	**0.1529**	0.7074

*Precision and recall are measured on the basis of the 5 × 5 × 5 blocks around the bifurcation points. Mean error and its corresponding standard deviation are measured in voxels away from the bifurcation points (not the 5 × 5 × 5 blocks)*.

*Best performing method in each metric are show in bold*.

## 4. Summary and Conclusions

We present DeepVesselNet, an architecture tailored to the challenges of extracting vessel networks and features using deep learning. Our experiments in sections 3.3 and 3.4 show that the cross-hair filters, which is one of the components of DeepVesselNet, performs comparably well as 3-D filters and, at the same time, improves significantly both speed and memory usage, easing an upscaling to larger data sets. Another component of DeepVesselNet, the introduction of new weights and the FP rate correction discussed in section 2.2, helps in maintaining a good balance between precision and recall during training. This turns out to be crucial for preventing over and under-segmentation problems, which are common problems in vessel segmentation. We also show from our results in section 3.4 that using sub-sampling layers in a network architecture in tasks which includes voxel-sized objects can lead to a fall in performance. Finally, we successfully demonstrated in sections 3.3 and 3.4 that transfer learning of DeepVesselNet through pre-training on synthetically generated data improves segmentation and detection results, especially in situations where obtaining manually annotated data is a challenge.

As future work, we will generalize DeepVesselNet to multiclass vessel tree task, handling vessel segmentation, centerline prediction, and bifurcation detection simultaneously, rather than in three subsequent binary tasks. We also expect that network architectures tailored to our three hierarchically nested classes will improve the performance of the DeepVesselNet. For example by using a multi-level activation approach proposed in Piraud et al. (2019) or through a single, but hierarchical approach starting from a base network for vessel segmentation, additional layers for centerline prediction, and a final set of layers for bifurcation detection.

The current implementation of cross-hair filters, network architectures and cost function are available on GitHub (Tetteh, 2019a). Datasets can also be downloaded from the wiki page of the same GitHub page.

## Data Availability Statement

The datasets presented in this study can be found in online repositories. The names of the repository/repositories and accession number(s) can be found at: https://github.com/giesekow/deepvesselnet/wiki/Datasets.

## Author Contributions

GT, VE, and MP contributed to experiments and writing of the manuscript. NF, MS, and JK contributed toward data generation and preparation. BW, CZ, and BM contributed toward experiment and manuscript verification, and supervisory role. All authors contributed to the article and approved the submitted version.

## Conflict of Interest

The authors declare that the research was conducted in the absence of any commercial or financial relationships that could be construed as a potential conflict of interest.
